# Relationship between Alexithymia and latent trigger points in the upper Trapezius

**DOI:** 10.1186/s13030-017-0116-6

**Published:** 2017-12-11

**Authors:** Hideaki Hasuo, Kenji Kanbara, Tetsuya Abe, Mikihiko Fukunaga, Naoko Yunoki

**Affiliations:** 1 Department of Psychosomatic Medicine, Kansai Medical University, Shinmachi 2-5-1, Hirakata, Osaka, 573-1090 Japan; 2 Department of Internal Medicine, Akaiwa Medical Association Hospital, Okayama, Japan

**Keywords:** Alexithymia, Depression, Latent trigger points, Upper trapezius, Manual examination

## Abstract

**Background:**

Latent trigger points (LTrPs) can be activated by future events, leading to pain. Few studies have reported LTrP risk factors. It has been suggested that alexithymia is associated with myofascial pain and diminished awareness of physical sensation. This study was designed to evaluate the relation between alexithymia and LTrPs found the upper trapezius of healthy individuals.

**Methods:**

The correlation between LTrPs and alexithymia, and between LTrPs and depression was analyzed in 160 healthy participants (80 male, mean age: 40.5 years [20 to 66 years]). Each participant was evaluated for potential LTrPs by careful manual examination and completed the Toronto Alexithymia Scale-20 (TAS-20) and the Beck Depression Inventory (BDI) to assess potential alexithymia and depressive symptoms, respectively.

**Results:**

LTrPs were observed in the upper trapezius of 76 participants (47.5%). TAS-20 scores were significantly higher in subjects with LTrPs than without LTrPs (*p* < 0.001); in contrast, there was no significant BDI score difference between these groups (*p* = 0.451). The LTrP risk for alexithymia was 2.74 (95% confidence interval [95% CI]: 2.10–3.58). There was no correlation between the TAS-20 and BDI scores (correlation coefficient: −0.04). Significant risk factors associated with LTrPs included the TAS-20 score (odds ratio [OR]: 1.11, 95% CI: 1.07–1.15) and age (OR: 1.05, 95% CI: 1.01–1.09).

**Conclusions:**

Alexithymia was associated with LTrPs in the upper trapezius of healthy individuals, suggesting that it may serve as a useful predictive factor.

**Trial registration:**

UMIN000027468. Registered 23 May 2017(retrospectively registered).

## Background

Trigger points (TrPs) are believed to play an important role in the development of myofascial pain. The definition of TrPs has evolved over time, and they are now described as hyperirritable nodules in the fascia located within a taut band of skeletal muscle fibers that, when palpated, are tender and produce referred pain sensations [1]. TrPs are classified as either active TrPs (ATrPs) or latent TrPs (LTrPs) [2, 3]. ATrPs generate spontaneous and recognized pain [2]. Although LTrPs are not responsible for spontaneous pain, they can produce local tenderness and/or transient referred pain sensations after manual examination [3].

LTrPs are found in many pain-free skeletal muscle fibers and can be activated by the continuous action of noxious stimuli; when this occurs, they essentially become ATrPs [4]. No standard therapy for the treatment of ATrPs has been established, highlighting the importance of preventive care. Indeed, it is critical for healthcare providers to be aware of the existence of LTrPs before they are activated and respond like ATrPs. The diagnosis of TrPs requires careful manual examination, which is considered a highly reliable procedure [5]. The upper trapezius has been the focus of most research on TrPs because of the high TrP prevalence in this area, in addition to providing easy access to the taut band [6, 7]. Experienced physiotherapists can reliably identify TrPs in the upper trapezius by palpation [8].

To better predict the presence of LTrPs, it is necessary to properly identify all potential risk factors. There are multiple known TrP risk factors, including ergonomic factors such as repetitive motion and structural factors such as scoliosis [9]. In addition, it has been reported that emotional factors such as anger and psychological distress, and psychosocial factors, such as insomnia, may constitute important TrP risk factors [10, 11]. In particular, there is strong evidence of a significant relationship between LTrPs and depressive symptoms in healthy subjects [12].

A relationship between myofascial pain and alexithymia has also been previously reported [10]. Alexithymia is a dimensional personality trait characterized by difficulty in expressing emotions and in identifying one’s own feelings and those expressed by others. The concept of alexithymia initially evolved from clinical observations of patients with psychosomatic disorders [13]. Alexithymia is considered to impede the successful regulation of emotions, particularly negative feelings, which results in a chronic sympathetic hyperarousal, an amplification of somatosensory sensations, and complaints concerning a variety of physical symptoms [14].

It has been reported that 32.5% of patients with chronic myofascial pain also receive the diagnosis of alexithymia, which positively correlates with pain severity [15]. In contrast, however, alexithymia has also been reportedly associated with a disorder of interoceptive sensation, which inhibits an individual’s awareness of their own physical sensations [16]. In light of these findings, we hypothesized that alexithymia might interfere with the awareness of muscular hypertonicity. This reduced awareness would then result in the formation of LTrPs, followed by the development of idiopathic myofascial pain. To our knowledge, no prior studies have investigated the relationship between alexithymia and LTrPs. Elucidating the relationship between alsexithymia and LTrPs may enable healthcare providers to justify careful manual examinations to search for possible LTrPs in patients with suspected alexithymia. It has been reported that patients with alexithymia have a low rate of help seeking; therefore, it is important for healthcare providers to become actively involved in the care of these patients [17].

The relationship between depression and both chronic pain and alexithymia has been previously investigated [15, 18]. In particular, myofascial pain is often comorbid with depression [19], whereas alexithymia, which is also substantially related to depression, may, in fact, predispose to it [14]. Finally, a relationship between LTrPs and depression has also been reported in healthy subjects [12]. Thus, because depression is a factor related to both alexithymia and LTrPs, a comprehensive study integrating all three factors is warranted.

The main objective of the present study was to investigate the relationship between alexithymia and LTrPs in the upper trapezius of healthy subjects.

## Methods

### Study design

This present study was designed as a cross-sectional survey of Japanese healthy individuals. Demographic information such as age, sex, and handedness, as well as measures of alexithymia and depression, were obtained from each participant. All participants had their upper trapezius evaluated by manual examination to identify LTrPs. When present, the distribution and the lateralization of LTrPs were also characterized.

The primary endpoint of the present study was the LTrP risk ratio for individuals with alexithymia.

### Participants

A total of 160 healthy subjects (80 male; mean age: 40.5 ± 11.2 years; age range: 20 to 66 years) participated in the study between July 2012 and December 2013. All participants were either employees at the Akaiwa Medical Association Hospital or were employee family members.

Three exclusion criteria were applied for participants: 1) the presence of chronic pain in the upper trapezius area, 2) a history of physical therapy or postoperative rehabilitation for the treatment of musculoskeletal disorders, and 3) a history of psychiatric treatment.

### Criteria used to diagnose LTrP

In the present study, TrPs were first diagnosed when the subject met all of the following four criteria: 1) the presence of a tender spot in a taut band; 2) the self-recognition of pain on a tender spot following palpation; 3) the presence of a predicted pain referral pattern (the pain distribution expected from a trigger point in the muscle); and 4) the existence of a local twitch response (transient local contraction of skeletal muscle fibers in response to palpation). These criteria, also known as Simons’ criteria, are essential for a proper TrP diagnosis [1, 20]. As previously stated, TrPs are divided into latent and active forms, with spontaneous pain deriving from ATrPs the primary difference between the two forms.

Based on a previous report [3], LTrPs were defined in the present study as the focus of hyperirritability in taut muscle bands, which is clinically associated with tenderness and/or referred pain distant from the LTrP site following manual examination.

### Measures: LTrP palpation procedure

Each participant was individually evaluated for LTrPs by palpation with the thumb. The palpation target area was limited to the both sides of the upper trapezius and did not include other scapular muscle groups such as the lower trapezius, supraspinatus, serratus anterior, and rhomboideus.

Based on a previous study, palpation was performed for each participant in a relaxed prone position while lying on a bed [12]. This position enabled us to easily distinguish the fibers of any taut bands placed under additional tension from other muscle fibers not under tension. The palpation area was approximately 3 cm wide (horizontal) and 2 cm in height (vertical). Once a taut band was identified, further palpation along the taut band was performed to search for local twitch responses. After the palpation search had been completed, we asked each participant if they felt any local or referred pain during manual compression. All palpation examinations were performed by one of two expert clinicians (H.H. and N.Y.), each with more than 10 years of experience in the diagnosis and treatment of myofascial pain.

### Measures: Self-report questionnaires

#### Toronto Alexithymia scale (TAS-20)

Each paticipant was evaluated for potential alexithymia symptoms based on the Japanese version of the Toronto Alexithymia Scale (TAS-20 total). The TAS-20 total is a self-report questionnaire consisting of 20 items with a rating scale of 1 to 5, which produces a total score ranging between 20 and 100. The TAS-20 total includes the following three subcategories: difficulty in identifying feelings (TAS-20 DIF, seven items), difficulty in describing feelings (TAS-20 DDF, five items), and externally oriented thinking (TAS-20 EOT, eight items). Adequate validity and reliability of the TAS-20 has been reported in previous studies [21, 22], and high construct validity and reliability have been established for the Japanese version [23, 24]. Based on the total score, the participants were classified into one of three groups: non-alexithymic (≤ 51), borderline (52 to 60) and alexithymic (≥ 61) [25].

#### Beck depression inventory (BDI)

Each participant was also evaluated for depressive symptoms with the Japanese version of the Beck Depression Inventory (BDI), which can be applied to both patients and the general population [26]. The BDI is a self-report questionnaire consisting of 21 items with a rating scale of 0 to 3, which produces a total score ranging between 0 and 63. Based on the BDI scoring method for the general population, participants were classified into one of two groups: depressive trend (≥ 21) and no depressive trend (≤ 20) [27].

### Statistical analysis

All group are reported as means with the standard deviation, 95% confidence intervals (95% CI), ranges, or frequency distribution (%), as appropriate. We used the unpaired t test for the following dependent variables: sex, age, TAS-20 score, and BDI score. Participants were classified into LTrP and non-LTrP groups. The relationship between LTrP and both the TAS-20 total score and the BDI score was analyzed with chi-square tests. The LTrPs risk ratio for alexithymia was determined. In addition, Pearson’s correlation coefficients between the TAS-20 total score, TAS-20 DIF score, TAS-20 DDF score, TAS-20 EOT score, and BDI score were calculated. The correlation coefficient between the TAS-20 total score and participant age was also determined. Lastly, a multivariate logistic regression analysis was performed with the presence of LTrP as the dependent variable, and sex (female), age, TAS-20 total score, and BDI score as the independent variables.

A *p*-value below 0.05 was considered statistically significant. Statistical procedures were conducted using SPSS version 18.0 J for Macintosh (SPSS, Inc. IBM, Chicago, IL).

## Results

The incidence of LTrPs (including the distribution of LTrPs) and sociodemographic information for all participants are shown in Table 1. LTrPs were observed in the upper trapezius of 76 participants (47.5%). There was no statistically significant sex or age difference between the LTrPs groups.

**Table 1:** Latent Trigger Point incidence and sociodemographic information

	LTrP incidence		*p*-value	Unilateral LTrP		Bilateral LTrP
	None LTrP	LTrP		DS	None DS	
Number of subjects	84	76		14	3	59
Sex, female	40	40	0.639	8	1	31
Age, years (means ± SD)	38.9 (12.4)	42.1 (10.9)	0.110	43.1 (10.8)	38.0 (9.0)	41.9 (11.1)

*SD* standard deviation, *LTrPs* latent trigger points, *DS* dominant side

Group means for the TAS-20 total, TAS-20 DIF, TAS-20 DDF, TAS-20 EOT, and BDI scores for both the LTrP and non-LTrP groups are shown in Table 2.

**Table 2:** Relationship between the presence of Latent Trigger Points and the mean scores of the Toronto Alexithymia Scale-20 and the Beck Depression Inventory

	Means(SD)		*p-value*	Unilateral LTrP		Bilateral LTrP
	None LTrP (*n* = 84)	LTrP (*n* = 76)		DS (*n* = 14)	None DS (*n* = 3)	(*n* = 59)
Total TAS-20	44.2 (11.2)	56.5 (10.9)	< 0.001	50.9 (7.7)	52.0 (5.3)	58.1 (9.8)
TAS-20 DIF	14.3 (4.3)	19.2 (4.4)	< 0.001	17.1 (4.0)	17.3 (2.0)	19.7 (4.5)
TAS-20 DDF	13.2 (4.3)	17.0 (4.4)	< 0.001	14.4 (4.6)	15.3 (3.2)	17.7 (4.3)
TAS-20 EOT	16.8 (5.4)	20.5 (4.5)	< 0.001	20.1 (3.3)	19.3 (2.5)	21.0 (4.6)
BDI	8.5 (6.3)	9.4 (7.4)	0.451	7.9 (8.3)	12.3 (13.7)	9.6 (6.9)

*SD* standard deviation, *DS* dominant side, *TAS* Toronto Alexithymia Scale, *DIF* Difficulty Identifying Feelings, *DDF* Difficulty Describing Feelings, *EOT* Externally Oriented Thinking, *BDI* Beck Depression Inventory, *LTrPs* latent trigger points

A test of independence between the presence of LTrPs and the TAS-20 score resulted in a Chi-square of 31.909 (*p* < 0.001), indicating that both factors are correlated with one another. In contrast, a test of independence between the presence of LTrP and the BDI score resulted in a Chi-square of 0.045 (*p* = 0.833), indicating that neither factors is correlated with the other. The TAS-20 total scores for both the LTrP and non-LTrP groups are shown in Table 3. Alexithymia was found for 38 participants (23.8%). The LTrP risk ratio in the alexithymic group versus non-alexithymic group was 2.74 (95% confidence interval [95% CI]: 2.10–3.58). The risk ratio for the absence of LTrP was 0.12 (95% CI: 0.04–0.36).

**Table 3:** Correlation between the Toronto Alexithymia Scale-20 score and the presence of Latent Trigger Points.

	LrTPs		Total
	Present	Not present	
Alexithymic group (TAS-20 total score: ≧ 61)	35	3	38
Non-alexithymic group (TAS-20 total score: <61)	41	81	122
(TAS-20 total score: > 51)	18	14	32
(TAS-20 total score: ≦ 51)	23	67	90
Total	76	84	160

*LTrPs* latent trigger points, *TAS* Toronto Alexithymia Scale

There was no correlation between any of the TAS-20 scores and the BDI score (Table 4). The TAS-20 total score was not correlated with age (correlation coefficient: 0.14). A logistic regression analysis identified two significant factors associated with LTrPs: the TAS-20 total score (odds ratio [OR]: 1.11, 95% CI: 1.07–1.15) and participant age (OR: 1.05, 95% CI: 1.01–1.09) (Table 5).

**Table 4:** Correlation coefficient between the Toronto Alexithymia Scale-20 and the Beck Depression Inventory score

	TAS-20 total	TAS-20 DIF	TAS-20 DDF	TAS-20 EOT	BDI
TAS-20 total		0.92^***^	0.77^***^	0.84^***^	− 0.04^n.s^
TAS-20 DIF			0.62^***^	0.71^***^	− 0.06^n.s.^
TAS-20 DDF				0.39^***^	- 0.07^n.s.^
TAS-20 EOT					0.01^n.s.^
BDI					

*LTrPs* latent trigger points, *TAS* Toronto Alexithymia Scale, *DIF* Difficulty Identifying Feelings, *DDF* Difficulty Describing Feelings, *EOT* Externally Oriented Thinking, *BDI* Beck Depression Inventory, *n.s* not significant

***p < 0.001

**Table 5:** Latent Trigger Points odds ratio for sociodemographic variables, alexithymia, and depression.

Variable	Coefficient β	SE	Wald	*P*-value	OR	95% CI
Sex, female	0.63	0.39	2.56	0.110	1.88	0.87–4.05
Age	0.05	0.02	8.61	0.003	1.05	1.01–1.09
TAS-20 total score	0.10	0.02	28.8	< 0.001	1.11	1.07–1.15
BDI score	0.03	0.03	1.47	0.226	1.03	0.98–1.09

*SE* standard error, *OR* odds ratio, *CI* confidence interval, *TAS* Toronto Alexithymia Scale, *BDI* Beck Depression Inventory

## Discussion

To our knowledge, this is the first study demonstrating relation between alexithymia and the presence of LTrPs in the upper trapezius of healthy participants. This finding is consistent with previous reports linking alexithymia and physical illness and supports the hypothesis that alexithymia interferes with the awareness of muscular hypertonicity, resulting in non-coping mechanisms [14].

More importantly, the study results indicate that alexithymia can be a critical predictive factor for LTrPs in the upper trapezius of healthy individuals. This finding further highlights the importance of conducting careful manual examinations of individuals with suspected alexithymia. In the LTrP group, mean values for all three alexithymia subcategories were significantly higher compared to those of the non-LTrP group. Thus, if an alexithymia trend is suspected for any of the three categories (difficulty in identifying feelings, difficulty in describing feelings, and externally oriented thinking), LTrPs should also be suspected. Our findings also identified age as another predictive factor of LTrPs; however, no correlation between age and the TAS-20 total score was observed, indicating that both factors are independent of one another. This result is consistent with the findings of a previous study also showing an absence of correlation between age and the TAS-20 score in healthy subjects [28].

The fact that LrTPs were present in 41 (33.6%) of 122 participants in the non-Alexithymic group suggests that there are multiple LTrP risk factors in addition to alexithymia. For instance, ergonomic factors such as the production of repetitive motions may increase the risk of unilateral LTrP. Although no statistically significant differences were observed, likely due to the small sample size of participant with unilateral LTrP (17 subjects), TAS-20 scores were nonetheless lower for the participants with unilateral LTrPs compared with those with bilateral LTrPs.

Another important finding of the present study is the absence of a relationship between LTrPs and the depressive symptoms of healthy subjects. Although depressive symptoms have been previously related to LTrPs [12], we did not find any evidence linking depression to LTrPs. This may be in part because the total number of LTrPs observed in each participant was not measured here, making it difficult to compare with previous findings linking BDI scores and the number of LTrPs [12]. A prior study reported that the average BDI score of healthy subjects with ≥ 5 LTrPs in the scapular muscle group (BDI score: 28.5) was significantly higher than the average for healthy individuals with no LTrPs (BDI score: 8.0) and the average for healthy individuals with ≤4 LTrPs (BDI score: 10.3). However, the number of LTrPs was determined based on the number of taut bands, a procedure that has yet to be properly validated. Furthermore, although the pathogenesis of TrPs has not yet been fully elucidated, a recent hypothesis suggests that they may result from a sensitized nociceptor in the epimysium [29]. Indeed, TrPs are found throughout the entire epimysium (plane), not in the intramuscular taut band (point). Subsequently, Rivers et al. [30] advocated that the essential diagnostic criteria for myofascial pain syndrome should exclude taut bands. The use of more objective measures acquired with tools such as tensiomyography and sonoelastography will allow investigators to more comprehensively examine the relationship between the TrPs and depression [31].

Furthermore, no correlation was observed between TAS-20 scores and BDI scores. It is possible that a different relationship between these measures exists for different alexithymia subtypes. Compared with type A, type B alexithymia is associated with greater difficulties in identifying feelings, and is also associated with a higher prevalence of self-reported major depressive disorder [32, 33]. The prevalence of type B alexithymia in healthy subjects may be low. However, in this study, it is difficult to evaluate alexithymia subtype because all three subcategory scores were high [34].

### Limitations

It should be noted that the present study does have limitations. First, although manual examination has been established as a reliable diagnostic procedure for the detection of LTrPs [5], the use of more objective diagnostic tools [31] such as tensiomyography and sonoelastography could be combined with manual examinations to further increase the reliability of the findings. Second, alexithymia was evaluated using self-report questionnaires. Although the TAS-20 has been validated [28], combining it with other measures such as those that could be obtained via a structured interview based on the Beth Israel Hospital Psychosomatic Questionnaire, might further increase diagnostic reliability [33]. Lastly, this was a single-facility investigation. Because the result of this research is limited to subjects with a low depression level, our results cannot be applied to patients with psychological or physical problems. Therefore, further investigations involving multiple study sites and various populations are warranted to confirm the present findings.

## Conclusions

Alexithymia was associated with the presence of LTrPs in the upper trapezius of healthy individuals, suggesting that it may serve as a useful predictive factor. This finding highlights the importance of careful manual examinations to detect LTrPs in individuals with suspected alexithymia. A future prospective cohort study is warranted to develop a better understanding of how alexithymia influences the progression of LTrPs to ATrPs.

## Abbreviations

95% CI: 95% confidence interval
ATrPs: Active trigger points
BDI: Beck depression inventory
LTrPs: Latent trigger points
OR: Odds ratio
TAS-20 DDF: Difficulty in describing feelings of the Toronto Alexithymia Scale-20
TAS-20 DIF: Difficulty in identifying feelings of the Toronto Alexithymia Scale-20
TAS-20 EOT: Externally oriented thinking of the Toronto Alexithymia Scale-20
TAS-20: Toronto Alexithymia Scale-20
TrPs: Trigger points
